# Barriers and enablers to guideline implementation strategies to improve obstetric care practice in low- and middle-income countries: a systematic review of qualitative evidence

**DOI:** 10.1186/s13012-016-0508-1

**Published:** 2016-10-22

**Authors:** Tim Stokes, Elizabeth J. Shaw, Janette Camosso-Stefinovic, Mari Imamura, Lovney Kanguru, Julia Hussein

**Affiliations:** 1 Department of General Practice and Rural Health, Dunedin School of Medicine, University of Otago, PO Box 56, Dunedin, 9054 New Zealand; 2 National Institute for Health and Care Excellence (NICE), Manchester, UK; 3 Munich, Germany; 4 The Institute of Applied Health Sciences, University of Aberdeen, Aberdeen, UK

**Keywords:** Systematic review, Qualitative synthesis, Framework synthesis, Guideline implementation, Obstetrics, Low- and middle-income countries

## Abstract

**Background:**

Maternal mortality remains a major international health problem in low- and middle-income countries (LMIC), and most could have been prevented by quality improvement interventions already demonstrated to be effective, such as clinical guideline implementation strategies. The aim of this systematic review was to synthesise qualitative evidence on guideline implementation strategies to improve obstetric care practice in LMIC in order to identify barriers and enablers to their successful implementation.

**Methods:**

We searched MEDLINE and CINAHL databases for articles reporting research findings on barriers and enablers to guideline implementation strategies in obstetric care practice in LMIC. We conducted a “best fit” framework synthesis of the included studies. We used an organisational “stages of change” model as our a priori framework for the synthesis.

**Results:**

Nine studies were included: all were based in Sub-Saharan Africa and in hospital health care facilities. The majority of studies (seven) evaluated one particular guideline implementation strategy: clinical audit and feedback (both criterion-based audit and maternal death reviews), and a minority (two) evaluated educational interventions. A range of barriers and enablers to successful guideline implementation was identified. A key finding of the framework synthesis was that “high” and “low” intrinsic health care professional motivation are overall enablers and barriers, respectively, of successful guideline implementation. We developed a modified “stages of change” model to take account of these findings.

**Conclusion:**

We have identified a number of quality improvement processes that are amenable to change at limited or no additional cost, although some identified barriers may be difficult to address without increased resources. We note the pathways to implementation may be complex and require further research to develop our understanding of individual and organisational behaviours and motivation in LMIC settings.

**Trial registration:**

PROSPERO CRD42015016062

**Electronic supplementary material:**

The online version of this article (doi:10.1186/s13012-016-0508-1) contains supplementary material, which is available to authorized users.

## Background

Maternal mortality remains a major international health problem. In 2010, 289,000 women died during and following pregnancy and childbirth. Almost all of these deaths occurred in low- and middle-income countries (LMIC), and most could have been prevented by interventions already demonstrated to be effective [1].

A key strategy to get effective interventions into routine clinical practice is to develop and implement evidence-based clinical practice guidelines [2]. In LMIC, deficiencies in obstetric care provision are commonly encountered, and clinical guidelines have been used to target specific areas of concern such as improving life-saving skills in obstetric emergencies, clinical management prior to emergency surgery and quality of perinatal care [3]. A range of guideline implementation strategies (for example, clinical audit and feedback) have been shown to be effective [2, 4] although the evidence base for LMIC is limited [3].

It is increasingly recognised that for guideline implementation strategies to be effective, we need to better understand why guideline implementation strategies work in some contexts and not in others [5] and also how we can ensure that they have an effect on quality improvement that lasts beyond the duration of any intervention studies (sustainability). We need to understand the context-specific barriers and enablers to implementation. This can be achieved through qualitative process evaluations of guideline implementation intervention trials [6, 7] and from evidence synthesis of qualitative research [8] on guideline/quality improvement implementation. The latter approach has been utilised in qualitative evidence synthesis of other comparable complex interventions: implementation of lay health worker programmes [7] and task-shifting in midwifery services [9].

The aim of this systematic review was to synthesise qualitative evidence on guideline implementation strategies to improve obstetric care practice in LMIC in order to identify barriers and enablers to their successful implementation. In addition, we undertook a complementary quantitative systematic review to determine whether strategies to promote the use of guidelines improve obstetric practices in LMIC [10].

## Methods

Using a registered protocol (PROSPERO: CRD42015016062), this review followed the methods as outlined below (Additional file 1).

### Review design

We undertook a qualitative systematic review. We chose to use a “best fit” framework synthesis approach [8, 11, 12] for two reasons. First, we hypothesised that the available data from existing qualitative studies in the study area would likely be “thin”—that is to say it would be limited in nature and descriptive in form—and thus not support an interpretive synthesis approach such as meta-ethnography [13]. Second, this approach is increasingly being used in quality improvement research [8] and allows one to identify and utilise existing frameworks for categorising barriers and enablers to quality improvement interventions in LMIC settings (e.g. SURE; organisational “stages of change”) [14, 15]. The “best fit” framework approach follows seven steps (Table 1) [8].

**Table 1:** Summary of “best fit” framework synthesis approach

Step 1	Define review question	Step 5	Create new themes by performing secondary thematic analysis on any evidence that cannot be coded into the a priori framework
Step 2	a) Systematically identify relevant primary research studies b) Identify relevant (“best fit”) publications of frameworks and conceptual models/theories	Step 6	Produce a new framework composed of a priori and new themes supported by the evidence
Step 3	Extract data on study characteristics from included studies and conduct study quality appraisal	Step 7	Revisit evidence to explore relationships between themes or concepts, in order to create a model
Step 4	Code evidence from included studies into the a priori framework identified in step 2		

Adapted from Booth and Carroll [8]

### Study inclusion criteria

#### Types of study methodology

*S*tudies that utilised qualitative methods for data collection and analysis were included. Mixed methods studies were eligible provided that it was possible to extract the findings derived from the qualitative research.

#### Types of studies and settings

Studies from LMIC (World Bank Definition: http://data.worldbank.org/news/new-country-classifications-2015) were included. Eligible practitioners were health professionals and paramedical professionals located in health facilities from tertiary to primary level and those working in primary care (e.g. auxiliary nurse midwives, clinical officers and medical assistants) in LMIC. The type of care targeted was all pregnancy care relating to antenatal, labour, delivery and the immediate postnatal periods for prevention, diagnosis, referral, treatment and general clinical management of obstetric complications.

#### Types of intervention

Eligible interventions included one of the following seven implementation strategies to change health care provider behaviour, either alone or in combination (terms and definition according to Cochrane Effective Practice and Organisation of Care (EPOC)—taxonomy http://epoc.cochrane.org/epoc-taxonomy):

1. Distribution of educational materials—published or printed, audio-visual materials—delivered personally or through mass mailings
2. Educational meetings, conferences, lectures, workshops or traineeships and training if provided in the context of evidence-based packages
3. Local consensus processes around identifying and agreeing important clinical issues and management approaches
4. Educational outreach visits, which could include feedback on provider performance
5. Local opinion leaders
6. Audit and feedback
7. Reminders, including obstetric protocols, checklists, diagnostic/decision flowcharts, or decision aids (Additional file 1)

### Exclusions

We excluded studies that:

- Only described current obstetric practice in LMIC, without specifically describing the barriers and enablers to the stated guideline implementation strategies
- Reported implementation at a health policy level
- Primarily targeted traditional birth attendants or untrained health and paramedical workers

We also excluded studies without abstracts.

### Search strategy and selection process (step 2)

#### Search strategy

We searched CINAHL (inception to September 2014) and MEDLINE (inception to April 2014). We utilised three published search filters in our search strategy: the Cochrane EPOC Group LMIC filter, designed to help identify studies relevant to low- to middle-income countries (http://epoc.cochrane.org/lmic-filters) and qualitative filters for use with CINAHL [16] and MEDLINE [17]. No date or language restrictions were applied. Through an iterative process, additional, qualitative research terms, used in studies meeting the inclusion criteria, were identified and employed alongside the qualitative filter. These two search elements were then combined with maternal mortality search terms and with implementation strategies search terms. Our aim was to identify as many relevant studies as possible and reduce the risk of missing potentially eligible studies (that is to maximise sensitivity rather than precision). See Additional file 2 for the full Medline strategy.

We also asked relevant content experts if they knew of additional relevant studies not already identified through the database searches.

### Study selection

Three review authors (TS, EJS and JCS) independently assessed the identified abstracts in pairs. Each reviewer’s list of included articles and accompanying rationale was compared with the list from the other reviewers and discrepancies discussed and resolved. Inclusion and exclusion criteria were also refined and clarified during this process. Where there was discrepancy the third reviewer was consulted. If there was still disagreement, then a fourth reviewer with content expertise (JH) was consulted. The full-text papers identified from abstract screening were also assessed for inclusion using the same approach.

### Quality assessment and data extraction

Four researchers (TS, EJS, MI and LK) independently assessed the quality of the included studies using the Critical Appraisal Skills Programme (CASP) quality assessment tool for qualitative studies (http://www.casp-uk.net/#!casp-tools-checklists/c18f8). No overall score or weighting was applied as the primary purpose of appraisal was to identify weaknesses in study design and how this may affect interpretation of the study findings, rather giving each study an overall score. No studies were excluded on quality alone.

### Data extraction and management (step 3)

We had intended to utilise, as other studies have done [7, 9], the SURE framework for assessing factors affecting the implementation of health system interventions [14]. However, we found this framework poorly fitted the themes presented in the included primary studies, in particular the process of delivering the intervention from planning its delivery to integration into routine practice. We instead utilised Bergh and Belizan’s organisational “stages of change” [15] model as our a priori framework [8] which we identified through the study literature search and which provided the “best fit” to the included studies. This organisational “stages of change” model was originally developed in LMIC for the implementation of kangaroo mother care (care of preterm infants carried skin-to-skin with the mother) [18] as a new health care intervention [19] by Bergh and colleagues in South Africa (1999–2006). The original model was termed a “progress monitoring model” and was conceptualised around three phases (pre-implementation, implementation and institutionalisation) and six constructs that depict a progression in implementation (awareness, adopting the concept, mobilisation of resources, evidence of practice, evidence of routine and integration, sustainable practice). This model was further refined by Belizan to explain barriers and facilitators to evidence-based perinatal care in Latin American hospitals [20] and by Belizan and Bergh to explain implementation of a perinatal audit programme in South Africa [15].

### Data synthesis (steps 4 to 7)

The lead reviewer (TS) constructed a data extraction template based on the organisational “stages of change” model [15] and coded the findings of the included studies into this framework (step 4) [8]. Three reviewers (EJS, JCS and JH) independently checked assignment of key findings from the included papers into the framework. A new theme was identified from the secondary thematic analysis, and this was utilised to explain why certain categories were “barriers” and “enablers” of quality improvement interventions (step 5) and a new framework (step 6) and hypothetical model generated (step 7).

## Results

### Overview of study settings and study types

**Fig. 1:**
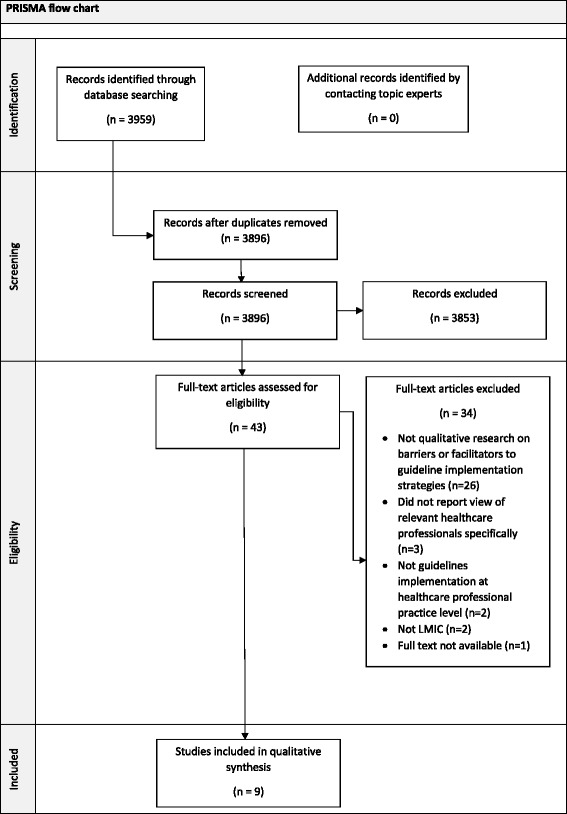
PRISMA flow diagram of search and exclusion process

Our search strategy yielded 3959 titles and abstracts. Of these, 9 studies met the inclusion criteria. The study selection process is detailed in a PRISMA flow chart (Fig. 1). Included studies were published between 2004 and 2012.

Study characteristics are described in Table 2. Included studies are referenced below in square brackets by their study ID (Table 2) and not according to citation number.

**Table 2:** Characteristics of included studies (*n* = 9)

Study details				Intervention	Intervention context	
Study ID	Author (year)	Data collection methods	Participants	Guideline implementation Strategy (intervention types presented in methods section)	Country	Setting
(1.)	Ameh et al. (2012) [35]	Questionnaire, focus group discussion (FGD), interviews	Midwives, doctors, midwifery and medical students (222 health care providers)	Educational intervention (intervention types a and b) using Cochrane reviews and UK RCOG Green Top guidelines through training for life saving skills in emergency obstetric care	Somalia (Somaliland)	Hospital and Community Clinics (all 5 regions of Somaliland)
(2.)	Belizan et al. (2011) [15]	FGD	Doctors, midwives, nurses (48 participants)	Audit and feedback (intervention type f) (Perinatal Problem Identification Programme (PPIP) an audit tool for the improvement of the quality of perinatal care in the public health care sector)	South Africa	Hospital (public health care sector)
(3.)	Dumont et al. (2009) [24]	Questionnaire, FGD, interviews, participant observation	Doctors (gynaecologist/obstetricians; other), midwives, paramedics (number of participants not stated)	Audit: maternal death reviews: “a qualitative, in-depth investigation of the causes and circumstances surrounding maternal deaths occurring at health facilities.” [36] (intervention type f)	Senegal	Hospital (5: 1 teaching/tertiary level; 1 district and 3 regional; number of maternity beds, range 33—120)
(4.)	Maaloe et al. (2012) [37]	Interviews	Assistant medical officer, nurse midwives (8 participants)	Audit (criterion-based) (intervention type f)	Tanzania	Hospital (2 rural mission hospitals with 200 beds each)
(5.)	Nyamtema et al. (2010) [38]	Questionnaire, Interviews	Members of maternal and perinatal audit committees and administrators (29 participants))	Audit (criterion-based): care compared against the national management guidelines for obstetric emergencies (intervention type f)	Tanzania	Hospital (4 major public hospitals and 4 major private hospitals in Dar es Salaam)
(6.)	Richard et al. (2008) [39]	Interviews	Doctors (gynaecologist/obstetricians; other), midwives (35 participants)	Audit (facility-based case reviews) [36] (intervention type f)	Burkina Faso	Hospital (26 bed obstetric unit in a district hospital in Ouagadougou)
(7.)	Smith et al. (2004) [40]	FGD, interviews	Labour ward staff (14 participants))	Educational intervention (better births initiative—targets practices where there is good evidence from systematic reviews of benefits or harm) [41] (intervention types a and b)	South Africa	Hospital (10 government maternity units in Gauteng)
(8.)	Van Hamersveld et al. (2012) [42]	Interviews, participant observation (of audit sessions)	Doctors (obstetrician; paediatricians; other), midwives (23 participants)	Audit (type of audit not specifically stated—includes critical incident audit/maternal death reviews) [36] (intervention type f)	Tanzania	Hospital (1 district hospital with approximately 5000 deliveries annually in Morogoro region)
(9.)	Hutchinson et al. (2010) [43]	Interviews	Doctors (obstetricians), midwives, nurse, social worker (8 participants) and Ministry of Health policy makers (2 participants)	Audit (near miss case reviews) [36] (intervention type f)	Benin	Hospital (5: 2 national university hospitals; 1 regional facility; 1 district hospital and 1 Catholic hospital. All located in different regions in southern Benin)

Of the 9 studies, all were based in Sub-Saharan Africa, 6 were set in low income countries (Somalia, Tanzania, Burkina Faso and Benin) (1,4,5,6,8,9), 1 in a lower middle-income country (Senegal) (3) and 2 in a upper middle-income country (South Africa) (2,7). All studies were based in hospital health care facilities, with one study including community health clinics (1). Study participants were medical practitioners (primarily obstetricians) and midwives.

The majority of studies [7] evaluated one particular guideline implementation strategy: clinical audit and feedback (both criterion-based audit and maternal death reviews) (intervention type f). Two studies used an educational intervention (intervention types a and b) (1,7). Further detail on included studies is provided in Table 2.

### Quality of the included studies

Included studies were either qualitative studies (2–9) or mixed method studies (1) which used either individual interview and/or focus group methodology. CASP appraisal revealed that these studies offered limited descriptions of the strategies used to select participants and the analysis used. When methods were stated, descriptive thematic analysis was undertaken. Detail was lacking for both the study context and the study findings in all studies. One study interpreted their findings using a theoretical model—Bergh and Belizan’s organisational “stages of change”—reported elsewhere in the literature (2) [15, 19]. One study (3) was conducted as part of a wider programme of implementation research to evaluate the effectiveness of maternal death reviews in sub-Saharan Africa [4, 21].

### Barriers and enablers to guideline implementation strategies to improve obstetric care practice (steps 4 to 6)

#### Pre-implementation phase

This phase (seven studies reported findings (1–4; 7–9)) refers to ways in which the organisation (hospitals) became aware of the need to improve the quality of obstetric care practices through quality improvement interventions [15].

Two key enablers were identified. The first was that the health care facility was more likely to initiate quality improvement if there was high intrinsic motivation [22]. A commitment to implementation was also seen as more likely where intrinsic motivation among staff was high and “social structures” (defined as good working relationships between staff) (7) existed to support and maintain practice (2,7,9). The second was a stated commitment from the organisation to implement the intervention—most commonly clinical audit; this was seen as strongest when there was a nominated lead person—a “driver”—who had the support of all levels of the organisation’s management tiers “from top to bottom” (2,3).

In contrast, the external imposition of quality improvement interventions such as clinical audit together with management having a “top down” approach may have contributed to lack of staff motivation to affect change (7,9). Two further barriers seen as inhibiting quality improvement initiation were both structural: a shortage of clinical staff and skilled birth attendants (1,4,8,9) and a shortage of essential equipment (e.g. vacuum extractors, magnesium sulphate) (1).

### Implementation phase

This phase (seven studies reported findings (2–4; 6–9)) refers to ways in which the quality improvement intervention was implemented, with a focus on the actual steps that need to be in place for a successful quality improvement programme [15]. This was the most commonly reported phase, and the specific barriers and enablers are fully set out in Table 3.

**Table 3:** Barriers and enablers to implementation phase of stages of change model [study ID]

Barriers	Enablers
Poor recording and extraction of clinical information Poor quality of information in medical records and collected information (3)	Good recording and extraction of clinical information
Data collection divided between numerous workers (3) Non-motivated data collector (3)	High level of qualifications/experience of data collector and appropriate training (2,3)
Audit meetings as a “blaming exercise” Audit meetings are a “blaming exercise” (2) run as a formal meeting where there is a fear of blame and punishment among attendees (fear of being judged/punished for findings; confidentiality not respected; afraid to tell story; may lie to protect oneself) (2,3,6,8,9) Case notes with deficiencies from medical doctors were not audited (“it’s not fair, only cases of midwives are audited, they have never chosen cases of the bosses. They do errors too”) (6)	Audit meetings as a “learning” exercise Audit meetings are run in an informal non-punitive learning environment which provides the opportunity for interaction, discussion and sharing of ideas about changing practice (2,7,8)
No local clinical leadership Audit only works when the one leader—Head of Department— is present. When s/he is not there no one else takes initiative, there is poor attendance at meeting and attendees are not motivated to participate as felt recommendations would not be implemented (4,8)	Local clinical leadership is crucial Local leadership (e.g. Head of Department) is a strong facilitator of clinical audit/maternal death review implementation and traditional hierarchical relationships may be an enabler. This occurs when the head of the hierarchy encourages a multidisciplinary approach and promotes staff acceptance of need to conduct audit (2,3,8,9)
Audit meetings are uni-professional Traditional medical hierarchies prevent the establishment of a multidisciplinary audit team. This excludes hospital managers and midwives/nurses who are then not motivated to take part in the audit (3,5,8)	Audit meetings are multi-professional Involvement of the whole multidisciplinary team was felt to motivate staff and promote implementation across the health system (2,7,9)
Poor communication of audit findings and feedback	Good communication of audit findings and feedback
Lack of feedback of recommendations to staff who did not participate, including management (3,6,8)	Findings and recommendations need to be communicated across the health system (2,3)

In terms of enablers, it is notable that local clinical leadership is a strong facilitator of clinical audit/maternal death review implementation, and traditional hierarchical relationships may be an enabler in certain situations.

In terms of barriers, a workplace culture characterised by low trust and low intrinsic motivation [22] may lead to the conduct of audit which is led by traditional hierarchies, occurs as a “blaming exercise” and findings not fed back to improve obstetric practice.

### Institutionalisation phase

Guideline implementation strategies such as clinical audit achieve this phase (four studies reported findings (2,5,7,9)) when the process of audit and feedback and acting on the results is integrated into routine practice and has been sustained for some time [15].

Institutionalisation was seen as more likely to occur where intrinsic motivation among staff was high, ownership of the audit was “very deep”(2) and social structures existed to support and maintain practice (2,7,9). In addition extrinsic motivation, such as financial assistance and reporting requirements to external agencies could also promote sustainability (9).

In contrast, a repeated failure to act on the findings of audits to prevent maternal death (e.g. evidence-based management of common conditions, closer monitoring and skilful management of labour) led to low intrinsic motivation of staff through staff becoming demoralised (5).

#### Revision to the stages of change model (steps 5 to 7)

**Fig. 2:**
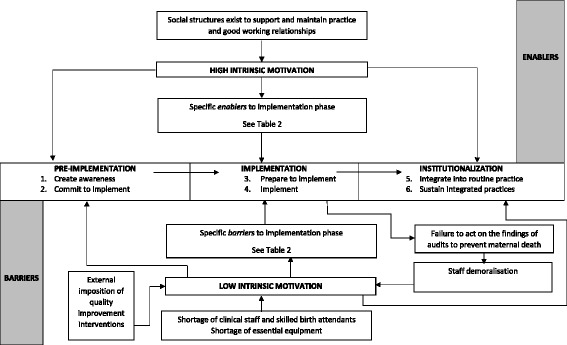
Revised “stages of change” model for implementation and sustainability of guideline implementation strategies in LMIC

Our secondary thematic analysis identified an important new theme of “motivation” [22] which cuts across the “stages of change” model and which may explain the identified barriers and enablers. It is also noted that the “stages of change” model per se does not specifically address barriers and enablers to implementation. We propose that the “stages of change” model can be modified [8] to include “high” and “low” intrinsic health care professional motivation as overall enablers and barriers, respectively, of the successful and sustainable implementation of clinical audit and feedback to improve obstetric care practice in LMIC. We present this modified framework in Fig. 2.

## Discussion

### Statement of principal findings

This qualitative systematic review of guideline implementation strategies to improve obstetric care practice in LMIC identified that the one implementation strategy which has been widely studied in this setting is clinical audit and feedback (both criterion-based audit and maternal death reviews) although educational interventions using evidence-based packages have also been studied. A range of barriers and enablers to successful guideline implementation was identified utilising an existing conceptual implementation framework (organisational “stages of change”) [15]. An important new finding, which allows modification of the “stages of change” framework (Fig. 2), is that “high” and “low” intrinsic health care professional motivation are overall enablers and barriers, respectively, of successful guideline implementation. Thus, a workplace culture characterised by low trust and low intrinsic motivation may lead to the conduct of strategies such as clinical audit which is led by traditional obstetric professional hierarchies, occurs as a “blaming exercise” and where the findings are not fed back to improve obstetric practice.

### Strengths and limitations of the review

The strength of this review is that it utilises a rigorous and systematic methodology of qualitative evidence synthesis. The underlying methodology—“best fit” framework synthesis—is being increasingly used to analyse and evaluate quality improvement interventions in health care [8, 11, 12]. Our hypothesis, that available data from included research studies would be limited in nature and descriptive in form (“thin” data), was correct, supporting our decision to use this methodology. In addition, we were able to identify a conceptual framework which best fitted the data—Bergh and Belizan’s organisational “stages of change” [15, 19]—and one which was significantly modified in the light of the qualitative synthesis. We also followed current best practice in relation to searching for and appraising the relevant qualitative evidence [7, 23].

A limitation of this review is the limited qualitative evidence available on the barriers and enablers to implementation strategies in obstetric care in LMIC. The number of studies that specifically reported barriers and enablers to implementation, as opposed to simply describing current obstetric practice, was small and limited to clinical audit and feedback (seven studies) and educational interventions using evidence-based packages (two studies). In addition, none of the included studies were conducted alongside an effectiveness intervention study, although one study (3) [24] was conducted as part of development work for subsequent development of a complex intervention trial [4]. This lack of qualitative research being carried out alongside trials of complex interventions has been noted by other researchers [25]. The current limited body of implementation research in this setting means that the revised “stages of change” model we have developed from the included studies (Fig. 2) is tentative and will benefit from further additions and refinement from future primary research. In particular, further exploration is required of the barriers and facilitators at each stage of the model and also whether the model can be seen as “linear”, or, if as Bergh and colleagues suggest, it “also allows for moving forwards and backwards; in other words, one step does not need to be fully completed before continuing with the next step, and hospitals can also regress in their implementation practices” [19].

This is the first study to qualitatively synthesise the evidence on barriers and enablers to guideline implementation strategies to improve obstetric care in LMIC. Its key finding—the importance of “high” and “low” intrinsic health care professional motivation as overall enablers and barriers, respectively, of implementation—is consistent with a recent systematic review of the influence of trust relationships on motivation in the health sector [22]. In summary, intrinsic motivation is positive, internalised, self-owned and promotes better task performance and higher competence; in contrast, extrinsic motivation is externalised, other-caused, low quality which does not consistently promote positive outcomes [26]. The systematic review found that, in common with other studies, low health worker intrinsic motivation (and attendant poor health worker practices) is common in LMIC. It also found, however, examples of high health worker intrinsic motivation in LMICs as well as in high-income countries [22]. Although we found that intrinsic motivation of staff was an important factor determining the uptake of guidelines, the studies reviewed did not provide analyses of the context and underlying causes affecting motivation. It would be conjecture on our part to draw any conclusion on how motivation is affected; however, others have identified underlying factors in LMIC such as resource availability, career progression and recognition as important [27]. The function and roles of women as part of the health workforce especially at primary and community level have also recently been put forward as an important consideration [28].

Since the cut-off date for the systematic review, a number of relevant primary qualitative studies have been published. These focus on maternal death review [29, 30] and guideline implementation [31] and have also identified the need to address similar barriers and enablers to those identified here.

## Conclusion

### Implications for health practice and policy

We have previously shown in our related quantitative systematic review that evidence of moderate to low level of quality suggests that interventions to implement guidelines may be effective in improving obstetric care in low- and middle-income countries [10]. In this study, we advance our understanding of the context in which such interventions are delivered by modifying an existing conceptual model for implementing quality improvement interventions in LMIC (“stages of change”) to emphasise the need for each level of the health system (from governmental policy down to individual patient—health professional encounters) [32] to have structures and processes in place which promote high intrinsic health care professional motivation. We have also shown the mechanisms by which low intrinsic motivation can act as a barrier to all stages of successful implementation. Further, we have drawn on the specific evidence from obstetric care practice in LMIC to assemble a clear set of specific enablers which need to be in place when implementing quality improvement interventions and also the converse: which specific barriers need to be addressed (Table 3). A number of identified barriers may be difficult to address without increased resources (e.g. shortage of essential equipment and staff) although a recent systematic review of the sustainability of health interventions in sub-Saharan Africa suggests that these barriers arise not only because of limited resources but also because of weak health systems, limited organisational capabilities and poor management of existing resources [33]. On the other hand, we have identified quality improvement processes (e.g. multidisciplinary meetings; local clinical leadership) that are amenable to change at limited or no additional cost. However, the pathways to change may be complex and involve understanding of individual and organisational behaviours and motivation. To explore these pathways effectively will require the prioritisation of high quality, in-depth qualitative research which incorporates multidisciplinary approaches from, for example, health psychology, organisational behaviours and the social sciences. Future effectiveness research evaluating guideline implementation strategies should aim to conduct a parallel process evaluation, in line with best practice when conducting complex intervention studies of guideline implementation [6, 10, 25, 34].

## Additional files

Additional file 1: PRISMA checklist. (DOC 61 kb)
Additional file 2: MEDLINE search strategy. (DOCX 27 kb)

## Abbreviations

LMIC: Low- and middle-income countries
