# Supplementary material for: Barriers and enablers to guideline implementation strategies to improve obstetric care practice in low- and middle-income countries: a systematic review of qualitative evidence
Source: Implement Sci. 2016 Oct 22;11:144. doi: 10.1186/s13012-016-0508-1 (PMC5075167; doi:10.1186/s13012-016-0508-1)
Supplement: Additional file 2: — MEDLINE search strategy. (DOCX 27 kb) [file 13012_2016_508_MOESM2_ESM.docx]

# MEDLINE search strategy

Database: Ovid MEDLINE(R) In-Process & Other Non-Indexed Citations and Ovid MEDLINE(R) <1946 to Present>

Search Strategy:

--------------------------------------------------------------------------------

1 *Reminder Systems/ (1260)

2 Reminder Systems/ (2151)

3 Decision Support Systems, Clinical/ (4677)

4 Physician's Practice Patterns/ (40380)

5 "Forms and Records Control"/ (7549)

6 exp Hospital Information Systems/ (26236)

7 exp Artificial Intelligence/ (51917)

8 exp Decision Making/ (118469)

9 "Appointments and Schedules"/ (6779)

10 exp Medical Records/ (84269)

11 exp Clinical Protocols/ (122364)

12 computer*.tw. (226346)

13 cellular phone/ (4161)

14 (mobile phone$1 or cell* phone$1 or txt or pxt or sms or mms).tw. (10613)

15 (remind$ or sticker$ or flowsheet$ or flow sheet$ or flowchart$ or flow chart$ or checklist$ or prompt$ or cue).tw. (137601)

16 (order adj1 (form$ or sheet$)).tw. (586)

17 ((request or encounter) adj1 form$).tw. (676)

18 ((tag or tagged or sticker or annot$) adj5 (note$ or record$ or sheet$ or chart$ or form$)).tw. (1842)

19 ((computer$ or information) adj3 feedback).tw. (1302)

20 ((decision or job) adj1 aid$).tw. (1799)

21 or/2-14 (659046)

22 or/15-20 (143535)

23 21 and 22 (11866)

24 1 or 23 (12282)

25 education, medical/ (47038)

26 education, professional/ or education, medical/ or education, nursing/ or education, pharmacy/ or education, public health professional/ (80396)

27 education, continuing/ or education, medical, continuing/ or education, nursing, continuing/ or education, pharmacy, continuing/ or education, professional, retraining/ (50147)

28 ((education$ or train$) adj2 (program$ or intervention$1 or meeting$1 or session$1 or strateg$ or workshop$1 or lecture$1 or symposi$ or course$1 or outreach)).tw. (84514)

29 ((education$ or train$) adj2 (pamphlet$1 or leaflet$1 or booklet$1 or poster$1 or brochure$1 or material$1 or bulletin$1 or handout$1 or hand out$1 or document$1)).tw. (5376)

30 ((education$ or train$) adj2 (clinical competenc$ or practice$ or health$ personnel or health care personnel or physician$1 or doctor$1 or clinician$1 or nurse$1 or provider$1 or practitioner$1 or professional$1 or midwife or midwives or birth attendant$1 or clinical officer$1 or medical assistant$1 or health$ worker$1 or health care worker$1 or obstetrician$1 or nursing or clinical)).tw. (59160)

31 guideline/ or practice guideline/ (24867)

32 Guideline Adherence/ (20007)

33 guidelines as topic/ or practice guidelines as topic/ (108963)

34 exp Evidence-Based Practice/ (59741)

35 ((guideline? or best practice? or evidence or EBM) adj2 (adher$ or apply$ or application or disseminat$ or implement$ or introduc$ or publication or release or uptake)).tw. (13499)

36 (evidence based adj2 (practice? or medicine or medical or treatment? or therap$ or healthcare or care)).tw. (22718)

37 (applied learning or knowledge transfer$ or knowledge translation).tw. (1718)

38 or/25-37 (440587)

39 opinion leader$.tw. (846)

40 exp Education/ (608888)

41 Professional Practice/ (14902)

42 Professional Role/ (8070)

43 professional$.tw. (174614)

44 education$.tw. (342698)

45 exp Leadership/ (26892)

46 opinion leader$.tw. (846)

47 influential*.tw. (10778)

48 or/40-44 (922838)

49 or/45-47 (38257)

50 48 and 49 (12624)

51 39 or 50 (13055)

52 exp Guidelines as Topic/ (111734)

53 Guideline Adherence/ (20007)

54 "Quality of Health Care"/ (56198)

55 Health Policy/ (49651)

56 Clinical Protocols/ (19640)

57 ((clinical or research or treatment) adj protocol$1).tw. (16168)

58 (standard$1 adj (care or quality)).tw. (4505)

59 exp Group Processes/ (145441)

60 exp Consensus Development Conference/ (9148)

61 (consensus adj (expert or local or develop$ or conference$1 or process$ or workshop$1)).tw. (6139)

62 (group adj (nominal or technique? or process$)).tw. (1881)

63 Delphi Technique/ (2658)

64 (delphi adj (techni$ or study or studies or approach$)).tw. (1586)

65 or/52-58 (258415)

66 or/59-64 (161494)

67 65 and 66 (14419)

68 (audit$ adj3 feedback).tw. (1815)

69 clinical audit/ or medical audit/ or "commission on professional and hospital activities"/ or nursing audit/ (18310)

70 management audit/ or benchmarking/ (12232)

71 feedback/ or feedback, psychological/ (28301)

72 "utilization review"/ or "concurrent review"/ or "drug utilization review"/ (10169)

73 Peer Review, Health Care/ (1277)

74 (audit or audits or auditing).tw. (25849)

75 (feedback or monitor*).tw. (593462)

76 (review adj3 record$1).tw. (10292)

77 chart review.tw. (21161)

78 (practice data or hospital$ data).tw. (3360)

79 benchmark$.tw. (17439)

80 exp Health Personnel/ (364628)

81 exp Hospitals/ (201084)

82 exp Professional Practice/ (215628)

83 Family Practice/ (59943)

84 professional competence/ or clinical competence/ (83059)

85 Physician's Practice Patterns/ (40380)

86 Nurse's Practice Patterns/ (880)

87 Quality Assurance, Health Care/ (48385)

88 "Quality of Health Care"/ (56198)

89 ((health$ personnel or health care personnel or physician$1 or doctor$1 or clinician$1 or nurse$1 or provider$1 or practitioner$1 or professional$1 or midwife or midwives or birth attendant$1 or clinical officer$1 or medical assistant$1 or health$ worker$1 or health care worker$1 or obstetrician$1 or nursing or clinical) adj3 (skill or skills or behaviour or behavior or competence or practice$1)).tw. (171368)

90 ((clinical or medical or private or general or family or professional or hospital?) adj practice?).tw. (164532)

91 (practice pattern? or pattern of practice).tw. (4752)

92 (quality adj (assurance or improvement or control)).tw. (58709)

93 (health care quality or healthcare quality or quality of healthcare or quality of health care or quality of care).tw. (35456)

94 performance.tw. (534034)

95 ((influenc* or chang*) adj3 (behaviour* or behavior*)).tw. (49587)

96 or/69-79 (701290)

97 or/80-95 (1630269)

98 96 and 97 (117206)

99 68 or 98 (118317)

100 Maternal Mortality/ (7734)

101 perinatal mortality/ (738)

102 Infant Mortality/ (24505)

103 Fetal Mortality/ (244)

104 Fatal Outcome/ (49576)

105 exp Pregnancy Complications/ (334172)

106 exp Pregnancy/ (704080)

107 exp Delivery, Obstetric/ (60589)

108 Maternal Welfare/ (5893)

109 exp Maternal Health Services/ (34202)

110 stillbirth$1.tw. (7405)

111 ((death$1 or mortalit$ or die or died or dying) adj10 (perinatal or newborn$1 or early neonatal)).tw. (17492)

112 ((death$1 or mortalit$ or die or died or dying) adj10 (pregnan$ or birth$ or childbirth$ or mother$ or matern$ or obstetric or antenatal or prenatal or antepartum or peripartum or postpartum or labor or labour or deliver$)).tw. (49075)

113 ((complication$1 or morbidit$ or emergenc$ or near miss$ or mortalit$) adj10 (pregnan$ or birth$ or childbirth$ or mother$ or matern$ or obstetric or antenatal or prenatal or antepartum or peripartum or postpartum or intrapartum or labor or labour or deliver$)).tw. (60025)

114 ((complication$1 or morbidit$ or emergenc$ or near miss$) adj10 (perinatal or newborn$1 or early neonatal)).tw. (8584)

115 or/100-114 (839433)

116 Developing Countries.sh,kf. (71837)

117 (Africa or Asia or Caribbean or West Indies or South America or Latin America or Central America).hw,kf,ti,ab,cp. (191829)

118 (Afghanistan or Albania or Algeria or Angola or Antigua or Barbuda or Argentina or Armenia or Armenian or Aruba or Azerbaijan or Bahrain or Bangladesh or Barbados or Benin or Byelarus or Byelorussian or Belarus or Belorussian or Belorussia or Belize or Bhutan or Bolivia or Bosnia or Herzegovina or Hercegovina or Botswana or Brasil or Brazil or Bulgaria or Burkina Faso or Burkina Fasso or Upper Volta or Burundi or Urundi or Cambodia or Khmer Republic or Kampuchea or Cameroon or Cameroons or Cameron or Camerons or Cape Verde or Central African Republic or Chad or Chile or China or Colombia or Comoros or Comoro Islands or Comores or Mayotte or Congo or Zaire or Costa Rica or Cote d'Ivoire or Ivory Coast or Croatia or Cuba or Cyprus or Czechoslovakia or Czech Republic or Slovakia or Slovak Republic or Djibouti or French Somaliland or Dominica or Dominican Republic or East Timor or East Timur or Timor Leste or Ecuador or Egypt or United Arab Republic or El Salvador or Eritrea or Estonia or Ethiopia or Fiji or Gabon or Gabonese Republic or Gambia or Gaza or Georgia Republic or Georgian Republic or Ghana or Gold Coast or Greece or Grenada or Guatemala or Guinea or Guam or Guiana or Guyana or Haiti or Honduras or Hungary or India or Maldives or Indonesia or Iran or Iraq or Isle of Man or Jamaica or Jordan or Kazakhstan or Kazakh or Kenya or Kiribati or Korea or Kosovo or Kyrgyzstan or Kirghizia or Kyrgyz Republic or Kirghiz or Kirgizstan or Lao PDR or Laos or Latvia or Lebanon or Lesotho or Basutoland or Liberia or Libya or Lithuania or Macedonia or Madagascar or Malagasy Republic or Malaysia or Malaya or Malay or Sabah or Sarawak or Malawi or Nyasaland or Mali or Malta or Marshall Islands or Mauritania or Mauritius or Agalega Islands or Mexico or Micronesia or Middle East or Moldova or Moldovia or Moldovian or Mongolia or Montenegro or Morocco or Ifni or Mozambique or Myanmar or Myanma or Burma or Namibia or Nepal or Netherlands Antilles or New Caledonia or Nicaragua or Niger or Nigeria or Northern Mariana Islands or Oman or Muscat or Pakistan or Palau or Palestine or Panama or Paraguay or Peru or Philippines or Philipines or Phillipines or Phillippines or Poland or Portugal or Puerto Rico or Romania or Rumania or Roumania or Russia or Russian or Rwanda or Ruanda or Saint Kitts or St Kitts or Nevis or Saint Lucia or St Lucia or Saint Vincent or St Vincent or Grenadines or Samoa or Samoan Islands or Navigator Island or Navigator Islands or Sao Tome or Saudi Arabia or Senegal or Serbia or Montenegro or Seychelles or Sierra Leone or Slovenia or Sri Lanka or Ceylon or Solomon Islands or Somalia or South Africa or Sudan or Suriname or Surinam or Swaziland or Syria or Tajikistan or Tadzhikistan or Tadjikistan or Tadzhik or Tanzania or Thailand or Togo or Togolese Republic or Tonga or Trinidad or Tobago or Tunisia or Turkey or Turkmenistan or Turkmen or Uganda or Ukraine or Uruguay or USSR or Soviet Union or Union of Soviet Socialist Republics or Uzbekistan or Uzbek or Vanuatu or New Hebrides or Venezuela or Vietnam or Viet Nam or West Bank or Yemen or Yugoslavia or Zambia or Zimbabwe or Rhodesia).hw,kf,ti,ab,cp. (2875086)

119 ((developing or less* developed or under developed or underdeveloped or middle income or low* income or underserved or under served or deprived or poor*) adj (countr* or nation? or population? or world)).ti,ab. (56323)

120 ((developing or less* developed or under developed or underdeveloped or middle income or low* income) adj (economy or economies)).ti,ab. (269)

121 (low* adj (gdp or gnp or gross domestic or gross national)).ti,ab. (146)

122 (low adj3 middle adj3 countr*).ti,ab. (3305)

123 (lmic or lmics or third world or lami countr*).ti,ab. (3299)

124 transitional countr*.ti,ab. (104)

125 or/116-124 (2980024)

126 exp Animals/ (17190582)

127 Humans/ (13285257)

128 126 not (126 and 127) (3905325)

129 review.pt. (1851827)

130 meta analysis.pt. (46020)

131 news.pt. (160273)

132 comment.pt. (574535)

133 editorial.pt. (350028)

134 cochrane database of systematic reviews.jn. (10298)

135 comment on.cm. (574533)

136 (systematic review or literature review).ti. (48364)

137 or/128-136 (6562198)

138 (interview or experience).mp. or qualitative.tw. (636192)

139 Qualitative research/ (18794)

140 (barrier* or facilitator*).tw. (168948)

141 (seek* adj3 care).tw. (7885)

142 (risk* adj2 (perception or perceived)).tw. (5863)

143 Process Assessment/ (2983)

144 Program Evaluation/ (45094)

145 Organizational Case Studies/ (9793)

146 (theme* or thematic).tw. (45391)

147 (assess* adj2 impact*).tw. (21008)

148 ethnograph*.tw. (5843)

149 (participant adj2 observ*).tw. (2757)

150 (observation* adj2 stud*).tw. (55885)

151 Pilot Projects/ (80819)

152 Focus Groups/ (16172)

153 focus group*.tw. (21566)

154 Interview*.tw. (218882)

155 Cross-Sectional Studies/ (170685)

156 Feasibility Studies/ (42082)

157 or/138-156 (1262782)

158 157 not 137 (1057526)

159 or/24,38,51,67,99 (564584)

160 115 and 125 and 158 and 159 (1593)

***************************
